# Preoperative simulation using three‐dimensional printer in four temporal bone surgeries

**DOI:** 10.1002/ccr3.7135

**Published:** 2023-05-12

**Authors:** Ichiro Fukumoto, Yukiyoshi Mita, Rie Shimmi, Yuri Sonobe, Tomohisa Iinuma, Kazuki Yamasaki, Shuji Yonekura, Toshimitsu Nemoto, Toyoyuki Hanazawa

**Affiliations:** ^1^ Department of Otorhinolaryngology/Head and Neck Surgery Chiba University Graduate School of Medicine Chiba Japan; ^2^ Department of Otorhinolaryngology Japanese Red Cross Narita Hospital Narita Japan

**Keywords:** otology, preoperative simulation, temporal bone surgery, three‐dimensional printer

## Abstract

Preoperative simulation using a three‐dimensional printer is effective to perform safe surgery by knowing the range limit of drilling in the temporal bone. Moreover, simulations using models are thought to be useful for education of young surgeon.

## 1 INTRODUCTION

The temporal bone is the most complicated site of the human body because it is difficult to understand its three‐dimensional anatomical structures and involves many vital organs such as the facial nerve, sigmoid sinus, middle cranial fossa, ossicular chain, cochlea, vestibule, and semicircular canals.^1^ Therefore, ear surgeries require extensive techniques, and a considerable number of years and experience are needed to become ear surgeons. In addition, having a prominent practiced hand in otology surgery is difficult and surgical opportunities for young doctors are decreasing, due to the increase in medical lawsuits. Consequently, many reports simulating surgery using the temporal bone and temporal bone size of the original model are available.^2^ However, institutions that allow doctors to perform practice courses using real temporal bone are limited because the procedure is legally complicated in Japan. Although temporal bone models have realistic designs, there is a difference in drilling bones during simulations, which is insufficient for surgical training.

Recently, several reported cases are available using three‐dimensional printers in the head and neck regions and during dental surgery, particularly mandibular bone surgery.^3^ In the surgical treatment of mandibular bone malignant tumors, particularly segmental resection of the mandibular bone, rigid reconstruction is necessary after tumor resection.^4^ Mandibular bone reproduction using a three‐dimensional printer is very useful because not only is the simulation of tumor resection possible but also the creation of the metal plate.

In 2016, additional surgical support can be calculated according to the size of the original organ solid model for tumor resection of various cancers in the head and neck regions in Japan. In our department, preoperative simulation was performed using a three‐dimensional printer for not only mandibular bone malignant tumor surgery but also maxillary malignant tumor surgery (maxillectomy, maxillary segmental resection), anterior skull base surgery through an endoscopic approach, middle ear surgery, and lateral skull base surgery. In this study, we reproduced the temporal bone using a three‐dimensional printer for middle ear and temporal bone surgeries preoperatively and reported the merits of our department.

## 2 MATERIALS AND METHODS

In four temporal bone surgeries, the temporal bone size of the original model was reproduced using a three‐dimensional printer preoperatively. We created DVD‐ROM copies of two‐dimensional 0.5‐ to 1.0‐mm computed tomography (CT) slice images that were constructed into three‐dimensional data using the software InVesalius 3 (Centro de Tecnologia da Informação Renato Archer [CTI], Brazil). Data were then converted into modeling data using slicing software Flash Print (Flashforge, Japan) and imported into Adventure 3 (Flashforge, Japan). All the models were made from a polylactic acid filament. Printing settings are as described below: lamination pitch 0.18 mm, filling rate 15%, hexagon filling pattern, print speed 60 mm/s, head temperature 210°C, platform temperature 50°C.

## 3 RESULTS

### 3.1 Case 1

Diagnosis: Squamous cell carcinoma of the external auditory canal (Pittsburgh classification T1).

Operative method: Lateral temporal bone resection.

**FIGURE 1:**
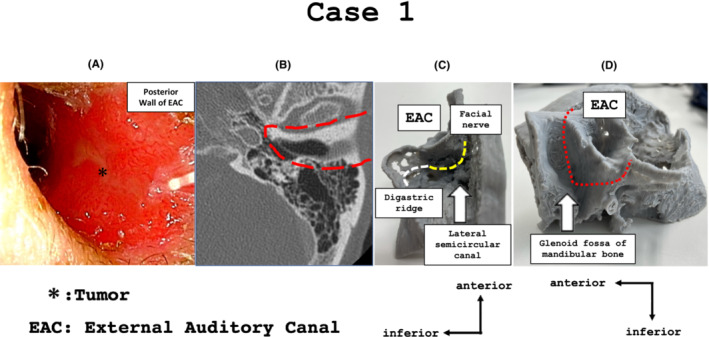
(A) An ulcerative lesion was observed from the posterior wall to the inferior wall of the left external auditory canal. (B) No obvious tumor was found on computed tomography (CT), and no bone destruction was observed. The dotted line is the resection line in CT axial scan. (C) We determined the depth of the antrum, lateral semicircular canals, middle cranial plate line, sigmoid sinus, digastric ridge, and facial nerve line. (D) The glenoid fossa of the mandible can be observed. Furthermore, the resection line can clearly be seen when we resect forward after performing mastoidectomy.

This is a case of case of a 68‐year‐old man who originally had a habit of cleaning his ears with a cotton swab. He visited the hospital with complaint of left ear pain and was diagnosed with squamous cell carcinoma by biopsy. The tumor was an ulcerative lesion from the posterior to the inferior wall of the left external auditory canal (Figure 1A). Bone destruction was not observed, and the diagnosis was T1 based on the Pittsburg classification (Figure 1B). The distance from the medial side of the tumor to the tympanic membrane was approximately 3 mm, preservation of the tympanic membrane was impossible, and the surgical policy was lateral temporal bone resection.

The temporal bone of the patient was reproduced using a three‐dimensional printer preoperatively. The point of reproduction was that we created a model which was completely performed mastoidectomy. In other words, we could identify the line of the middle cranial fossa, sigmoid sinus, posterior wall of the external auditory canal, and the depth of the lateral and posterior semicircular canals. The digastric ridge was also clearly reproduced in this model, and the facial nerve line between the anterior portion of the digastric ridge and lateral semicircular canals was confirmed (Figure 1C). In addition, the excision line that progressed from the superior and inferior walls to the anterior wall of the external acoustic canal was able to proceed according to the preoperative simulation (Figure 1D).

### 3.2 Case 2

Diagnosis: Squamous cell carcinoma of the external auditory canal (Pittsburgh classification T3).

Operative method: Subtotal temporal bone resection.

This is a case of a 52‐year‐old woman who visited the hospital with complaint of left otorrhea. A tumor in the right external auditory canal was observed and biopsied, resulting in a diagnosis of squamous cell carcinoma.

**FIGURE 2:**
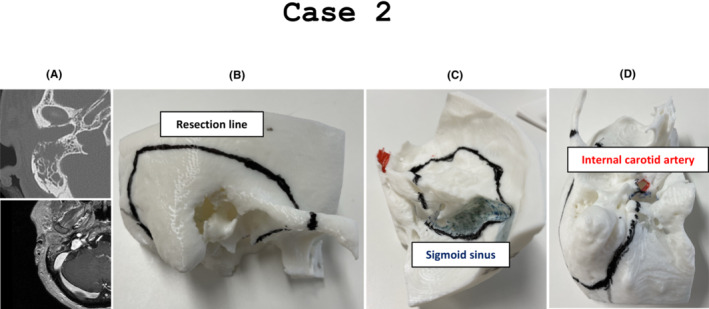
(A) Case 2 was diagnosed with Pittsburgh classification T3 because the tumor invaded into the mastoid and tympanic cavities. (B–D) Because it was planned to perform a subtotal temporal bone resection through the middle cranial fossa, the model was created in which mastoidectomy was not performed, unlike that in case 1. The model was painted blue for the sigmoid sinus line and red for the internal carotid artery line. The resection line is shown as the black line.

The tumor had progressed to the mastoid and tympanic cavity and was diagnosed with Pittsburgh classification T3. In this case, we decided to perform a lateral temporal bone resection once. However, the tumor was protruding in the mastoid and the tympanic cavity; therefore, it was considered that lateral temporal bone resection could not be performed without cutting into the tumor (Figure 2A). Hence, we decided to perform subtotal temporal bone resection together with neurosurgeons using the middle cranial fossa approach. In this case, a full‐scale model was created using a three‐dimensional printer before surgery. Since the surgery utilized the middle cranial fossa approach, unlike that in case 1, we created a model that did not require mastoidectomy (Figure 2B). In addition, the model was colored to indicate the lines of the sigmoid and internal carotid arteries (Figure 2C, D). Preoperatively, we consulted with the neurosurgeons to confirm the excision line (indicated by black line). Because it was an anatomically unfamiliar approach for otolaryngologists, the discussion was very useful for preoperative simulation. Finally, the tumor could be resected margin‐free.

### 3.3 Case 3

Diagnosis: Right cholesteatoma (Pars flaccida type, Stage II).

Operative method: Tympanoplasty type 0 with mastoidectomy (canal wall down).

**FIGURE 3:**
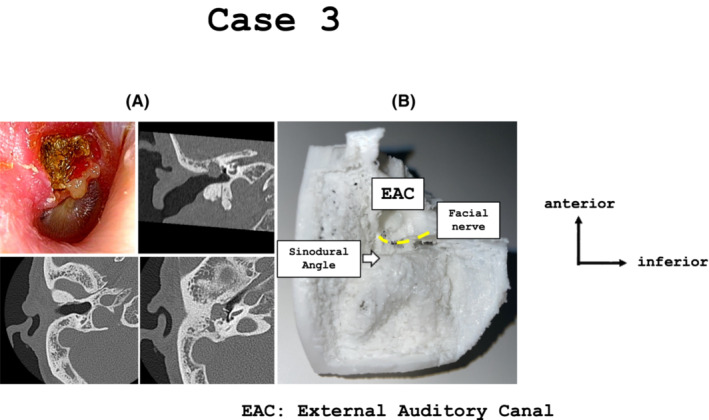
(A) In case 3, tympanoplasty type 0 and canal wall down mastoidectomy were performed for right flaccid‐type cholesteatoma, extending from the attic to the mastoid cavity. (B) A full‐scale model was created for the mastoidectomy. The facial ridge was created, as low as possible because canal wall down mastoidectomy would be performed. Additionally, the lines of the sinodural angle and protruding sigmoid sinus can be observed in this model.

A 72‐year‐old man presented with complaints of right otorrhea and was diagnosed with right cholesteatoma and otorrhea. Preoperative CT revealed a cholesteatoma extending from the attic to the mastoid. Destruction of the malleus and incus was observed, but cholesteatoma did not extend around the stapes (Figure 3A); however, tympanoplasty type 0 and mastoidectomy (canal wall down) were adopted.

We reproduced a temporal bone model that were performed mastoidectomy completely. Because of the canal wall down mastoidectomy, the posterior wall of the external auditory canal was drilled, and the facial ridge was made, as low as possible (Figure 3B). The risk of facial nerve damage was reduced by confirming the facial ridge line before surgery. In this case, the sigmoid sinus protruded into the mastoid, and the jugular bulb was at a relatively high position. Although the cholesteatoma had advanced deeply into the sinodural angle, preoperative simulation confirmed the lines of the middle cranial fossa and sigmoid sinus, making it possible to remove the cholesteatoma without damaging the vital structures.

### 3.4 Case 4

Diagnosis: Left cholesteatoma (Pars flaccida type, Stage II).

Operative method: Tympanoplasty type III with mastoidectomy (canal wall down).

**FIGURE 4:**
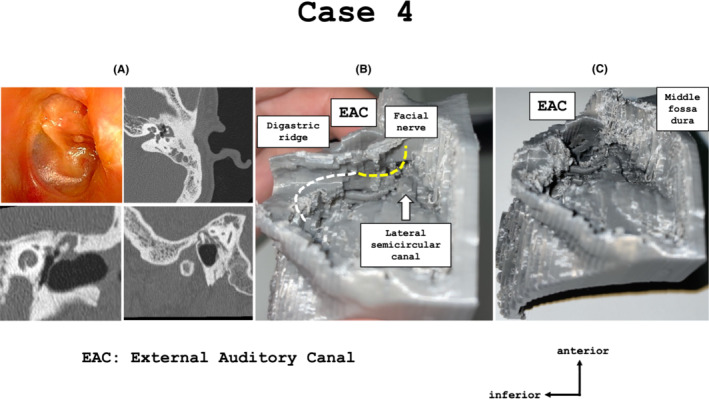
(A) In case 4, tympanoplasty type III and canal wall down mastoidectomy were performed for left flaccid‐type cholesteatoma. The computed tomography sagittal view showed that the middle fossa plate was lower towards the anterior, and the cholesteatoma extended to the mastoid and supratubal recess from the attic. (B) A full‐scale model that was performed the canal wall up mastoidectomy was created. We confirmed the line of the sigmoid sinus, posterior wall of the external acoustic canal, digastric ridge, facial nerve, and lateral semicircular canal. (C) We created the model that was used to performed the canal wall down mastoidectomy. We confirmed that the anterior of the middle fossa plate was lower.

A 37‐year‐old man presented with left otorrhea and was diagnosed with stage II left pars flaccida‐type cholesteatoma. Preoperative CT images revealed a cholesteatoma extending from the attic to the mastoid and supratubal recess (Figure 4A). Additionally, in the sagittal CT view, the middle fossa plate was lower toward the anterior; therefore, it is important to secure the anterior visual field during surgery. First, we reproduced a temporal bone model that were performed canal wall up mastoidectomy completely (Figure 4B). The posterior wall of the external auditory canal, sigmoid sinus, crest of the digastric ridge, and line of the facial nerve were confirmed. However, the anterior line of the middle fossa plate and supratubal recess could not be confirmed; therefore, it was considered insufficient for surgical simulation. Thus, we removed the posterior wall of the external auditory canal and created a full‐scale model which were performed canal wall down mastoidectomy (Figure 4C). We simulated the line of the middle cranial fossa preoperatively and removed the cholesteatoma that had extended into the supratubal recess without damaging the middle fossa dura.

## 4 DISCUSSION

Malignant tumors occurring in the temporal bone are rare with 1.3 per 1 million individuals affected, and are said to be approximately 0.2% of all head and neck cancers.^5^ Among cancers, external auditory canal cancers are the most common, and fewer cancers develop from the middle ear and ceruminous gland.^6^ Squamous cell carcinoma was the most common histologic type in 39% of cases, followed by basal cell carcinoma in 14%, and adenoid cystic carcinoma in 7%.^7^

Treatment of external auditory canal cancer includes surgery, radiotherapy, and chemotherapy, as well as common cancer therapy, and requires combined modality therapy.^8^, ^9^, ^10^ For the surgical treatment of external auditory canal cancer, temporal bone segmental resection, lateral temporal bone resection, and subtotal temporal bone resection are cited.^11^ These surgeries are technically similar to otology surgery, and the degree of difficulty is high; however, they are often performed in authorized cancer institutions. With regard to head and neck surgeons, anatomical knowledge of temporal bone surgery is required; however, there are few temporal bone surgeons in cancer therapy institutions. Based on these findings, we believe that a safe surgical strategy for external auditory canal cancer is necessary.

Recently, several reports of surgical simulation using three‐dimensional printers have been available^12^ in temporal bone surgeries.^13^ Additionally, it is useful not only in the simulation of surgery but also in surgery education.^14^ In our department, practice courses using actual temporal bones are sometimes held, but the opportunities are limited. In surgical education, drilling the original model using a three‐dimensional printer is important,^15^ but there is a difference in drilling simulated bone from real temporal bone.

In 2016, additional surgical support was calculated for temporal bone tumors based on the size of the original solid organ model for tumor resection in Japan. In our department, preoperative simulations using a three‐dimensional printer for temporal bone tumors, such as external acoustic canal cancer, were performed in almost all cases.

When performing lateral temporal bone resection in cases with Pittsburgh classification T1 or T2, as in case 1, mastoidectomy should be performed initially; however, mastoidectomy should be performed, as much as possible to secure the anterior field of view of the external acoustic canal. Preoperative simulations are advantageous because few surgeons have trained in ear surgeries at cancer specialty facilities. Case 2 had a Pittsburgh classification of T3 because the cancer invaded the mastoid and infiltrated the middle ear cavity. In previous reports, T3 cases had worse prognosis than T1 or T2 cases ^16^, ^17^ and were indicated for subtotal temporal bone resection, which may cause postoperative cranial nerve palsy.^7^ Moreover, postoperative margin‐positive cases have a worse prognosis than negative cases.^18^ In such cases, securing a tumor‐free surgical margin is challenging. In cases where the tumor slightly extends to the tympanic cavity or protrudes into the anterior parotid gland or temporomandibular joint, tumor‐free surgical margins may be obtained through lateral temporal bone resection. In case 2, it was impossible to create a tumor‐free surgical margin through lateral temporal bone resection, and subtotal temporal bone resection was performed using the middle cranial fossa approach. The full‐scale model created before the operation was meaningful in sharing the image of the operation with the neurosurgeon.

For otology surgeries, such as tympanoplasty and mastoidectomy, we created the models preoperatively. Several patients with otitis media or cholesteatoma have poor mastoid pneumatization, and mastoidectomy is difficult to perform in these patients. Furthermore, in cases such as case 3, in which the sigmoid sinus protrudes to the mastoid cavity, surgeons are hesitant to perform posterior excision, and the surgical field becomes restricted, making it difficult to perform superior‐quality surgery. The simulation was satisfactory in showing and visualizing the excision limit before surgery in such cases. Moreover, when performing canal wall up mastoidectomy, the posterior wall of the external auditory canal was as thin as possible. During the canal wall down mastoidectomy, the posterior wall of the external acoustic canal was drilled, and the facial ridge was created, as low as possible.

In this study, we created simple and inexpensive models; therefore, we could not reproduce the auditory ossicles. However, the use of three‐dimensional printers for ossicular chain reconstruction has also been reported.^19^ Additionally, by creating full‐scale models made from the material to be drilled, we can expect higher‐quality preoperative simulations.^14^

Creating full‐scale models that are useful preoperatively is beneficial. Additionally, it was considered useful to share preoperative images with other doctors using the created full‐scale models and to educate young doctors and medical students during surgery.

## 5 CONCLUSION

Cases of middle ear and temporal bone surgeries using a three‐dimensional printer were examined. The risk of complications could be reduced by knowing the range limit of drilling and the position of the vital organ. Moreover, simulations using models are thought to be useful for the education of young doctors and medical students.

## AUTHOR CONTRIBUTIONS

**Ichiro Fukumoto:** Writing — original draft. **Yukiyoshi Mita:** Investigation. **Rie Shimmi:** Writing — review and editing. **Yuri Sonobe:** Writing — review and editing. **Tomohisa Iinuma:** Writing — review and editing. **Kazuki Yamasaki:** Writing — review and editing. **Shuji Yonekura:** Writing — review and editing. **Toshimitsu Nemoto:** Writing — review and editing. **Toyoyuki Hanazawa:** Project administration.

## CONFLICT OF INTEREST STATEMENT

The authors have no conflicts of interest to declare.

## CONSENT

Written informed consent was obtained from the patient to publish this report in accordance with the journal's patient consent policy.

## Data Availability

The data that support the findings of this study are available from corresponding author upon reasonable request.
